# The course of the radial nerve in the distal humerus: A novel, anatomy based, radiographic assessment

**DOI:** 10.1371/journal.pone.0186890

**Published:** 2017-10-26

**Authors:** H. P. Theeuwes, B. van der Ende, J. W. Potters, A. J. Kerver, J. H. J. M. Bessems, G-J. Kleinrensink

**Affiliations:** 1 Department of Neuroscience-Anatomy and Erasmus MC Anatomy Research Project (EARP), Erasmus MC, University Medical Center, Rotterdam, The Netherlands; 2 Department of Surgery, VieCuri Medical Center, Tegelseweg BL Venlo, The Netherlands; 3 Department of Orthopedic Surgery, Reinier de Graaf Gasthuis, Reinier de Graafweg AD Delft, The Netherlands; 4 Department of Anesthesiology, Erasmus MC, University Medical Center, Rotterdam, The Netherlands; 5 Department of Surgery, Franciscus Gasthuis & Vlietland, Kleiweg PM, Rotterdam, The Netherlands; 6 Department of Orthopedic Surgery, Erasmus MC, University Medical Center, Rotterdam, The Netherlands; Georgia Regents University, UNITED STATES

## Abstract

Iatrogenic nerve injury during fracture surgery of the upper arm is a well-known complication. Prevention of this type of injuries would be of great value. The literature describes several methods to reduce this type of injury, but no perfect solution is at hand. In this study we introduce a new radiographic evaluation of the course and variation of the radial nerve in the distal part of the humerus in relation to bony landmarks as observed on a plain (trauma) radiographs. Aim of this new approach is to reduce the chance of iatrogenic nerve injury by defining of a danger zone in the distal upper arm regarding the radial nerve and hence give an advise for future implant fabrication.

**Methods and findings:**

Measurements were done on both arms of ten specially embalmed specimens. Arms were dissected and radiopaque wires attached to the radial nerve in the distal part of the upper arm. Digital radiographs were obtained to determine the course of the radial nerve in the distal 20 cm of the humerus in relation to bony landmarks; medial epicondyle and capitellum-trochlea projection (CCT). Analysis was done with ImageJ and Microsoft Excel software. We also compared humeral nail specifications from different companies with the course of the radial nerve to predict possible radial nerve damage.

**Results:**

The distance from the medial epicondyle to point where the radial nerve bends from posterior to lateral was 142 mm on AP radiographs and 152 mm measured on the lateral radiographs. The average distance from the medial epicondyle to point where the radial nerve bends from lateral to anterior on AP radiographs was 66 mm. On the lateral radiographs where the nerve moves away from the anterior cortex 83 mm to the center of capitellum and trochlea (CCT). The distance from the bifurcation of the radial nerve into the posterior interosseous nerve (PIN) and superficial radial nerve was 21 mm on AP radiographs and 42 mm on the lateral radiographs (CCT).

**Conclusions:**

The course of the radial nerve in the distal part of the upper arm has great variety. Lateral fixation is relatively safe in a zone between the center of capitellum-trochlea and 48 mm proximal to this point. The danger zone in lateral fixation is in-between 48–122 mm proximal from CCT. In anteroposterior direction; distal fixation is dangerous between 21–101 mm measured from the medial epicondyle. The more distal, the more medial the nerve courses making it more valuable to iatrogenic damage. The IMN we compared with our data all show potential risk in case of (blind) distal locking, especially from lateral to medial direction.

## Introduction

Humeral shaft fractures account for 3–5% of all skeletal fractures and 20% of all humeral fractures[1]. Humeral fractures are due to a fall (87%), motor accidents (8%) or direct trauma (5%)[2]. 90% of humeral shaft fractures are treated *conservatively* and have good clinical outcome. Surgical intervention occurs in 10% of the fractures where surgery can either be early in open, pathological or comminuted fractures or delayed [3]. Both in conservative and surgical treatment there is a risk of soft tissue injury where neurovascular structures are especially at risk. Radial nerve palsy occurs in 11% of humeral shaft fractures due to direct trauma or temporary entrapment before fracture reposition [4, 5]. As opposed to ulnar or median nerve palsy, which are very rare as described in literature only by some case reports or cadaveric studies [6–8]. The severity of the radial nerve palsy varies from symptoms of sensory disturbance of the dorsolateral aspect of the hand to complete paralysis of the brachioradial muscle and the extensor muscles of the wrist and fingers. On average 88% of radial nerve palsies recover completely. 81% of these cases resolve spontaneously in an average period of six months. The other 19% require surgical re-intervention [8]. Ten to twenty percent of all radial nerve palsies due to humeral shaft fractures is iatrogenic and is caused by manipulation or dissection during surgical treatment [5].

The intraoperative risks of radial nerve damage depends on the surgical method. Plate osteosynthesis results in high union rates but requires extensive dissection of soft tissue with a high risk of nerve injury. Alternatively, the fracture can be stabilized by intra-medullary nailing (IMN) in which soft tissue surrounding the fracture is preserved and the chance of nerve damage is reduced. During IMN procedures the radial nerve is injured in one to six percent. The most common mechanism of injury to the radial nerve occurs during distal locking, in particular when using lateral-to-medial directed locking screws. Both plate osteosynthesis and IMN are comparable in terms of functional outcome and rates of union [9, 10].

Several anatomical studies describe the course and position of the radial nerve in relation to anatomical landmarks such as the lateral inter muscular septum, the triceps aponeurosis or bony landmarks such as the medial or lateral epicondyle. These structures serve as reliable perioperative landmarks for the course of the radial nerve [11–13]. However, no studies are known in which the position of the radial nerve was determined pre-operatively on a radiograph. In order to assess the course of the radial nerve in the distal humeral region, anatomical landmarks such as the acromion can be used. In clinical situations however, it is much more reliable to assess the position of the radial nerve by using the medial epicondyle and capitellum-trochlea line (in lateral view) as landmarks.

The aim of this study is to reduce the chance of iatrogenic injury to the radial nerve in the distal upper arm by determine the course and variation of the radial nerve. This was done in relation to anatomical bony landmarks; epicondyle and center of capitellum-trochlea, as observed on a plain (trauma) radiographs and hereby determine a ‘danger zone’ during lateral and AP fixation in which the radial nerve is at risk and hence give an advise for future implant fabrication.

## Materials and methods

For this study human specimens were used. All humans whom donated their body for science used in our cadaver study had a written form of consent. Regarding the Dutch law and our institutional ethics committee, no other forms or documentation is necessary.

Twenty arms were dissected in the supine position, derived from ten Caucasian embalmed human anatomic specimens (6 female and 4 male). Mean age was 72 years (range 57–89). All specimens were embalmed at room temperature between 24 and 48 hours post-mortem. Embalming was performed according the AnubiFiX^®^ (AnubiFiX, Rotterdam, The Netherlands) method, preserving tissue and joint flexibility thereby providing a representative anatomy and mechanics [14–16].

**Fig 1:**
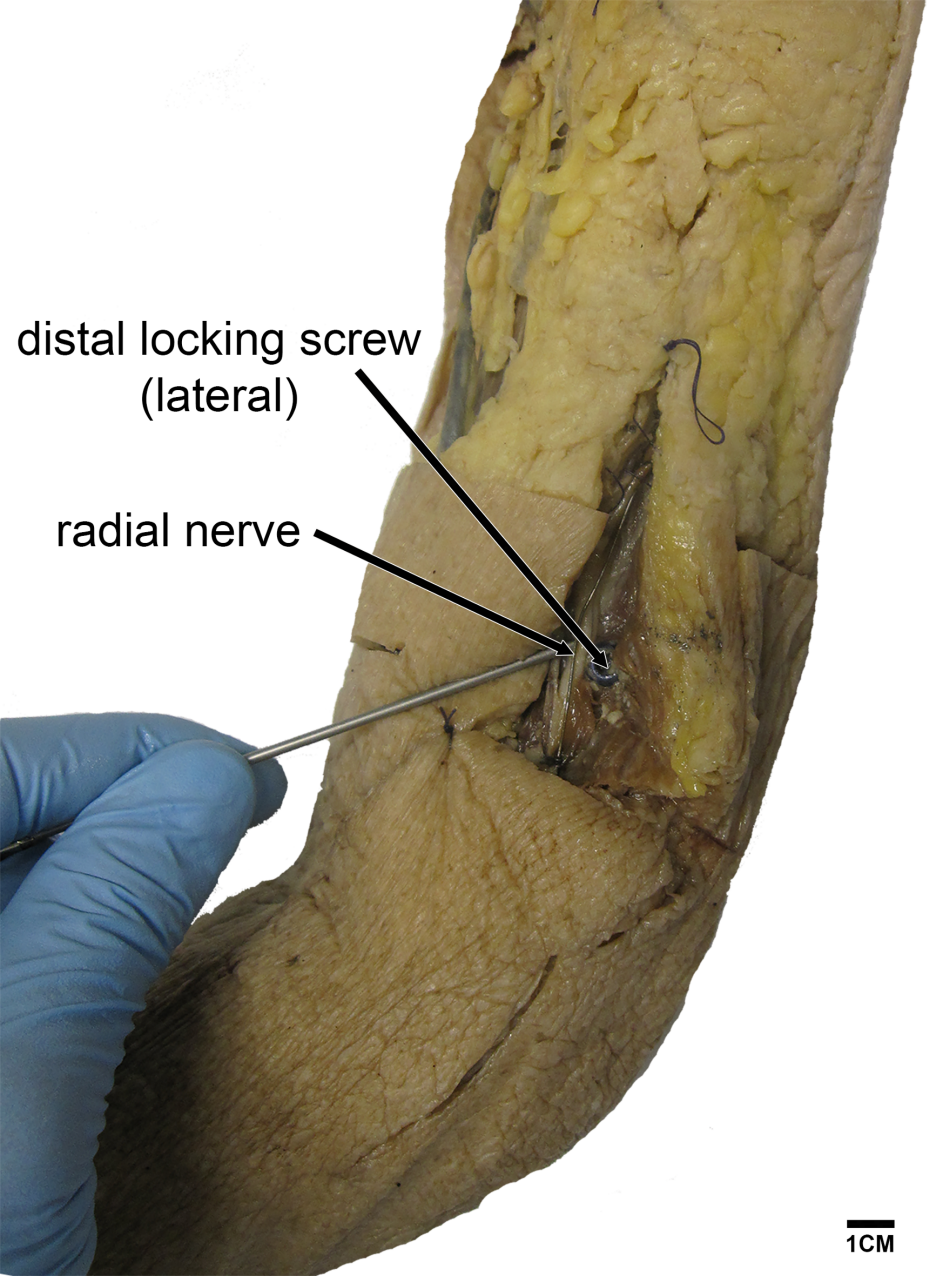
Left dissected humerus. In one arm we inserted a T2 humerus nail showing potential risk of damaging the radial nerve during distal screw fixation (lateral to medial direction).

**Fig 2:**
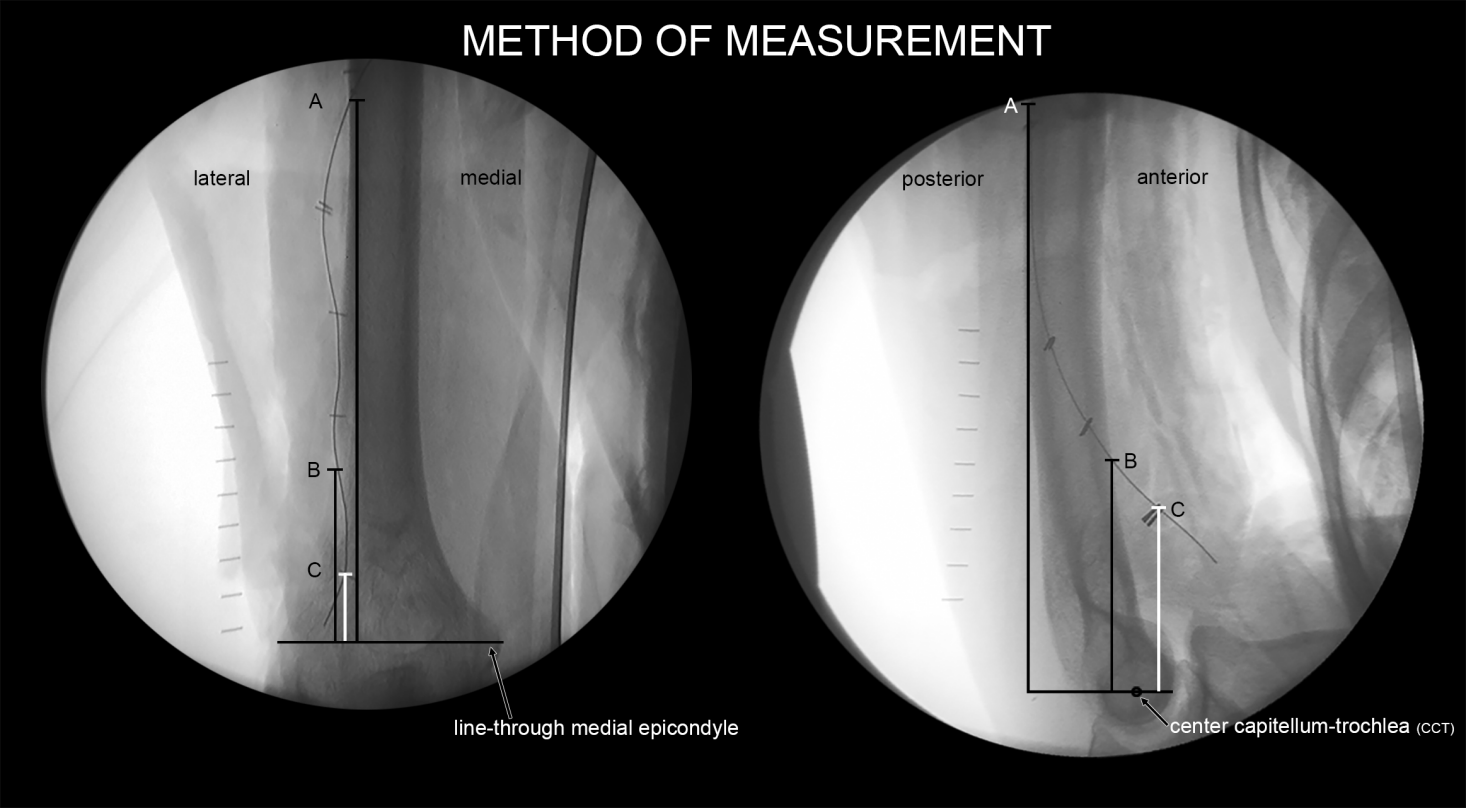
Radiographs (AP and lateral view) of prepared upper arm showing a marked radial nerve and method of measurements. Points of interest A, B and C are marked.

A direct lateral distal humerus incision was made and the triceps, brachial and brachioradial muscles were retracted. The radial nerve was identified in the distal part of the upper arm and exposed. Care was taken not to dissect the nerve and its surrounding tissue to keep normal anatomical relations of the nerve intact. A radio-opaque wire was placed directly adjacent to the radial nerve and attached with 4 metal clips (Figs 1 and 2). The bifurcation of the radial nerve in the superficial and deep branch was marked with two close adjacent clips. The fascia was then closed keeping the marked radial nerve in its anatomical position. Subcutaneous and superficial layers were closed with conventional continuous sutures. Radiographs with a calibration bar were obtained with a mobile C-arm system (BV 29, Philips, Eindhoven, The Netherlands). Lateral radiographs were directed through capitellum and trochlea (figure of 8) to obtain a true lateral image. Anteroposterior (AP) radiographs were directed perpendicular to this axis, shown in Fig 2. After scaling by using the calibration bar, measurements were performed on the standardized digital radiographs using ImageJ (version 1.38), Fig 2 [17]. Distances on AP radiographs were measured between points of interest (POI) and the line perpendicular to the humeral shaft running through the medial epicondyle and on true lateral radiographs from the center of capitellum-trochlea projection (CCT). POI are point A representing the location where the radial nerve courses from the dorsal to the lateral side of the arm. The second POI (B) locates the radial nerve bending from lateral to anterior in AP view and away from the anterior cortex in lateral view. The bifurcation of the radial nerve into the superficial and deep branch was marked as point C, Fig 2. The distance of the radial nerve to the lateral cortex on AP radiographs was measured in a 10 mm interval and the distance to the dorsal cortex on lateral radiographs is measured in a 20 mm interval.

Nail specifications from the three most commonly used intramedullary nails in the Netherlands were obtained; Synthes^®^, Stryker^®^ and Smith&Nephew^®^. These specifications were compared to our data. A ‘danger zone’ was defined by measuring the position of the proximal ridge of the olecranon fossa in comparison to the medial epicondyle and CCT. Next, 20 mm was added to correct for the ideal position of the humerus nail (20 mm proximal to the proximal ridge of the olecranon fossa). With these data, the position of the distal locking hole(s) as it would be in an *in vivo* situation can be correlated to the measured location of the radial nerve in AP and lateral obtained radiographs. Data were analyzed using Microsoft Excel^®^ version 14.7.2 (Microsoft^®^ Excel^®^ for Mac 2011).

## Results

All measurements performed on the radiographs are presented in Table 1.

**Table 1:** Data overview of measurements on AP and lateral humeral radiographs with Mean, Standard Deviation and Range in cm.

**Anteriorposterior view**									
**No**	**Spec No**	**Side**	**Gender**	**Age (Y)**	**Position x-ray**	**humerus length**	radial nerve curves **posterior to lateral** in relation to medial epicondyle	radial nerve **lateral to anterior** in relation to medial epicondyle	**split radial nerve** in profunda/superficialis in relation to medial epicondyle
1	393	L	M	81	AP	37	16,23	6,93	2,14
2	393	R	M	81	AP	37,2	out op pic	10,05	3,76
3	396	L	F	64	AP	31,1	out op pic	4,40	1,86
4	396	R	F	64	AP	31,5	out op pic	5,52	3,39
5	401	L	M	89	AP	31,4	11,75	5,56	3,05
6	401	R	M	89	AP	32,4	out op pic	7,63	2,5
7	405	L	F	86	AP	34,5	16,52	7,39	1,59
8	405	R	F	86	AP	35,4	14,1	8,04	2,5
9	411	L	F	63	AP	34,5	12,36	6,45	1,33
10	411	R	F	63	AP	35,5	out op pic	7,19	3,34
11	492	L	M	65	AP	30	12,10	5,55	0,76
12	492	R	M	65	AP	30	12,84	5,51	2,98
13	522	L	M	64	AP	33	15,50	6,50	0,53
14	522	R	M	64	AP	33	15,96	6,87	1,29
15	574	L	F	57	AP	33	15,62	3,55	-0,42
16	574	R	F	57	AP	33	12,42	3,74	-0,41
17	582	L	M	85	AP	35	12,91	2,11	0,51
18	582	R	M	85	AP	35	11,90	2,49	3,48
19	584	L	F	64	AP	34	16,05	6,09	2,77
20	584	R	F	64	AP	34	16,22	7,56	4,87
N							**15**	**20**	**20**
	**mean**			**71,80**		**33,53**	**14,17**	**6,63**	**2,09**
	**SD**			**11,61**		**2,07**	**1,88**	**1,96**	**1,43**
	**min**			**57,00**		**30,00**	**11,75**	**2,11**	**0,42-**
	**max**			**89,00**		**37,20**	**16,52**	**10,05**	**4,89**
**Lateral view**									
**No**	**Spec No**	**Side**	**Gender**	**Age (Y)**	**Position x-ray**	**humerus length**	radial nerve curves **posterior to lateral** in relation to CCT	radial nerve away **from anterior cortex** to CCT	**split radial nerve** in profunda/superficialis in relation tot CCT
1	393	L	M	81	LAT	37	18,16	7,15	3,93
2	393	R	M	81	LAT	37,2	19,83	6,17	5,67
3	396	L	F	64	LAT	31,1	17,04	4,75	5,41
4	396	R	F	64	LAT	31,5	14,4	5,07	3,68
5	401	L	M	89	LAT	31,4	13,56	11,33	5,76
6	401	R	M	89	LAT	32,4	12,44	7,30	4,10
7	405	L	F	86	LAT	34,5	16,75	7,90	4,46
8	405	R	F	86	LAT	35,4	14,79	9,66	5,44
9	411	L	F	63	LAT	34,5	15,10	6,25	3,49
10	411	R	F	63	LAT	35,5	16,29	8,07	6,07
11	492	L	M	65	LAT	30	12,77	5,89	0,66
12	492	R	M	65	LAT	30	11,32	12,17	3,35
13	522	L	M	64	LAT	33	out op pic	8,08	0,51
14	522	R	M	64	LAT	33	16,34	8,33	1,67
15	574	L	F	57	LAT	33	14,23	11,91	0,47
16	574	R	F	57	LAT	33	out op pic	6,44	0,98
17	582	L	M	85	LAT	35	15,96	10,98	0,41
18	582	R	M	85	LAT	35	16,93	10,79	4,11
19	584	L	F	64	LAT	34	14,11	9,80	3,13
20	584	R	F	64	LAT	34	13,11	7,60	4,00
N							**18**	**20**	**20**
	**mean**			**71,80**		**33,53**	15,17	8,28	4,16
	**SD**			**11,61**		**2,07**	2,18	2,28	1,94
	**min**			**57,00**		**30,00**	11,32	4,75	0,41
	**max**			**89,00**		**37,20**	19,83	12,17	6,07

**Fig 3:**
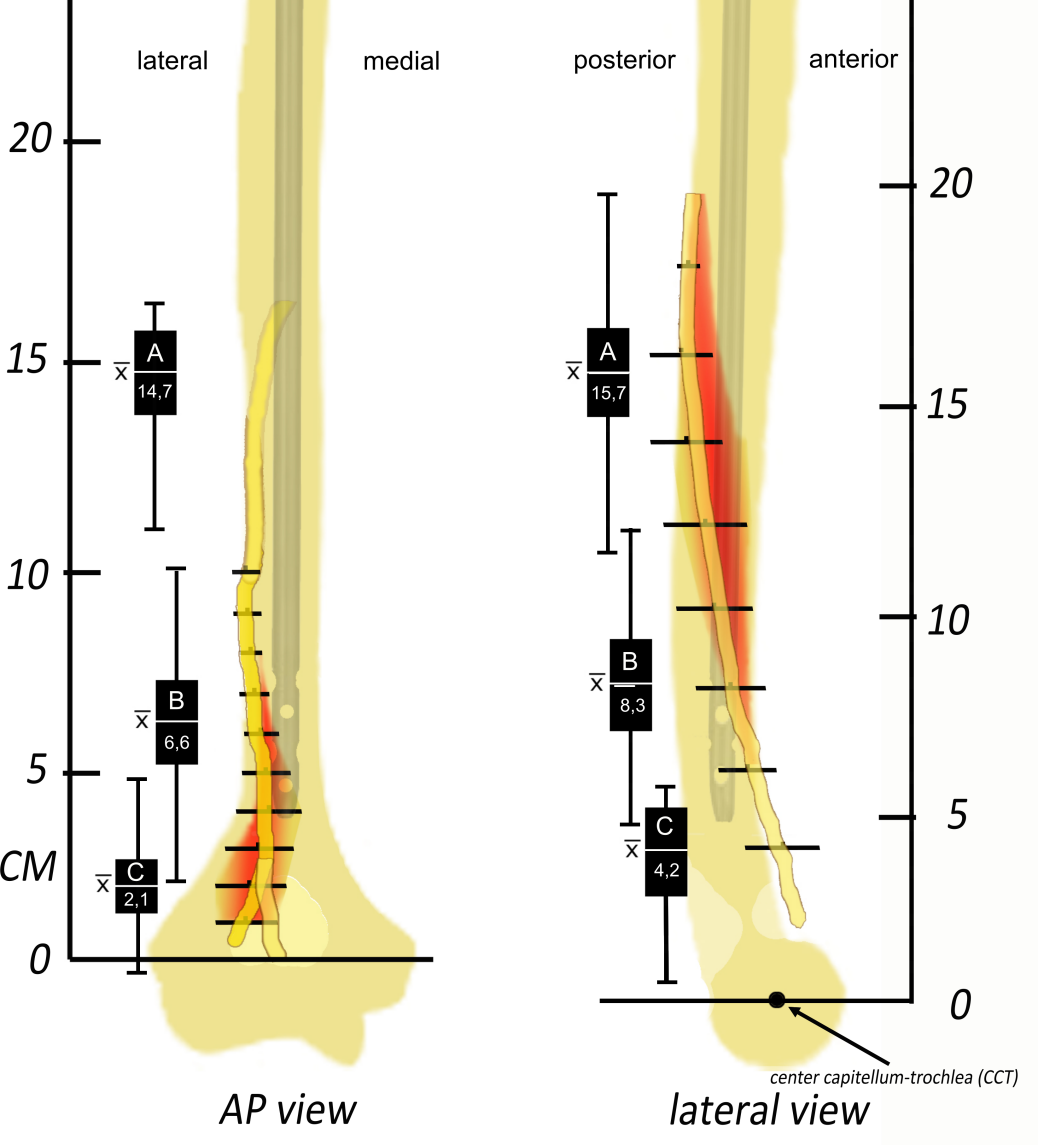
Graph representing the course of the radial nerve and the ‘danger zones’. The red area in the AP view marks the ‘danger zone’ for AP fixation. The red area in the lateral view shows the ‘danger zone’ for lateral drilling (horizontal lines are the mean ± 2SD, red area represents range of possible radial nerve location.

Data is represented as mean (± SD) and range, unless otherwise stated. Average humerus length was 335 mm (SD 21; 300–372 mm) from the tip of the humerus to the medial epicondyle. No significant differences were found between humeral length and gender (Student *t-*test, *p* = 0.8). The average distance from the medial epicondyle to point where the radial nerve bends from posterior to lateral was 142 mm (SD 19; 118–165) on AP radiographs and 152 mm (SD 22; 113–198 mm) measured on the lateral radiographs to the center of capitellum and trochlea (CCT). The average distance from the medial epicondyle to point where the radial nerve bends from lateral to anterior on AP radiographs was 66 mm (SD 20; 21–101). On the lateral radiographs POI B refers to the point where the nerve moves away from the anterior cortex 83 mm (SD 23; 48–122) to the center of capitellum and trochlea (CCT). The distance from the bifurcation of the radial nerve into the posterior interosseous nerve (PIN) and superficial radial nerve was 21 mm (SD 14; -41–49) on AP radiographs and 42 mm (SD 19; 4–61) on the lateral radiographs (CCT). Fig 3 shows the average course of radial nerve in relation to both the lateral cortex (AP Fig) and the posterior cortex (lateral Fig) of the distal humerus, POI A, B and C are marked.

The Expert nailing system (Synthes®) and TRIGEN Long Bent Humeral Nail (Smith&Nephew®) have AP distal locking holes whereas the T2-PHN (Stryker®) has both AP and lateral distal locking holes. When placed anterograde and in a correct manner, 20 mm proximal from the olecranon fossa ridge, the Expert nailing system on average locks AP between 48 and 78 mm from the medial epicondyle and locks lateral at 63 mm. TRIGEN locks AP between 44 and 54 mm. T2-PHN locks AP between at 44 mm and lateral between 52 and 62 mm. When placed retrograde and correct, Expert-nailing system locks distally AP between 34 and 68 mm and T2-PHN locks lateral between 46 and 72 mm, as is presented in Table 2. No retrograde intramedullary humerus nail by Smith&Nephew^®^ is available. All distal locking holes were in a position in which the radial nerve is at risk when locking the nail.

**Table 2:** Overview of position of locking holes in long humeral intramedullary locking nails from Synthes®, Stryker® and Smith&Nephew® Distance is measured from the most distal end of the nail, to the medial epicondyle in AP direction and to the center of capitellum and trochlea in lateral view. The range in which distal locking can be performed, measured in mm.

**Anteroposterior view**		
	mean range	holes
**Synthes®** anterograde	48–78	3
retrograde	34–68	3
**Stryker®**	44	1
**Smith&Nephew®**	44–54	2
**Lateral view**		
	mean range	holes
**Synthes®** anterograde	63	3
**Stryker®** anterograde	52–62	2
retrograde	46–72	3
		distance in mm

## Discussion

This study investigates the course of the radial nerve in relation to landmarks observed on standard radiographs. Various anatomical studies determine the course of the radial nerve in order to avoid iatrogenic injury during surgical procedures [12, 13, 18, 19]. In contrast to previous studies we used radiological guidance to determine the location of the radial nerve rather than surface anatomy [20].

We observed significant variation in the position of the radial nerve in the distal third part of the humerus. Lateral fixation or pin placement is relatively safe in a zone between the center of capitellum-trochlea (CCT) and 48 mm proximal of this point. The danger zone in lateral fixation is in-between 48 and 122 mm proximal from CCT. In anteroposterior direction; distal fixation remains dangerous between 21 and 101 mm measured from the medial epicondyle. The more distal, the more the nerve moves toward the medial side, Fig 3, making it more valuable to iatrogenic damage. Besides the radial nerve, structures such as the median nerve, brachial artery and vein are also at risk during distal locking. We therefore advocate blunt dissection to the bone and usage of a drill protector to limit the risk of iatrogenic nerve damage. Pre-operative skin marking with radiographic aid can decrease the chance of damaging the radial nerve.

Noger et al. illustrated the potential damage of the radial nerve by insertion of a distal locking nail when placing a Synthes® standard unreamed humerus nail [21]. This study further explores this potential complication by describing the average course of the radial nerve and its orientation in relation to the distal humerus and the potential risk of radial nerve damage in two more humerus nail systems. Due to natural occurring anatomical race and gender differences the course of the radial nerve can even vary more [22]. The one-third, two-thirds ratio as described by Fleming et al. was measured from the acromion to the lateral epicondyle [12]. We advocate that the sub acromial space negatively influences the measurement of the humerus as this space varies when the deltoid muscle is completely relaxed (e.g. under local or general anesthesia). Therefore no ratio comparison could be made. Small numbers of specimens and lack of *in situ* measurements of different intramedullary nails and their distal locking places in reference to the radial nerve location are factors that limit this study. Despite these factors, the data can very well be applied to clinical settings. The most important message is the awareness of potential radial nerve damage during surgical placement of any distal locking screw or external fixators in lateral or AP direction. We would recommend manufacturers of intramedullary implants to use this information in future implant development.

## Conclusions

Based on our results we conclude that the course of the radial nerve in the distal part of the upper arm has great variety. Lateral fixation or pin placement is relatively safe in a zone between the center of capitellum-trochlea (CCT) and 48 mm proximal of this point. The danger zone in lateral fixation is in-between 48 and 122 mm proximal from CCT. In anteroposterior direction; distal fixation is dangerous between 21 and 101 mm measured from the medial epicondyle. The more distal, the more the nerve moves toward the medial side, Fig 3, making it more valuable to iatrogenic damage. The IMN we compared with our data all show potential risk in case of (blind) distal locking, especially from lateral to medial direction.
